# Comparison of accuracy of anterior and superomedial approaches to shoulder injection: an experimental study

**DOI:** 10.1051/sicotj/2015044

**Published:** 2016-03-25

**Authors:** Bancha Chernchujit, Nutthapon Zonthichai

**Affiliations:** 1 Department of Orthopaedic Surgery, Faculty of Medicine, Thammasat University Hospital Klong-Nueng, Klong-Luang, Pathumthani 12120 Thailand

**Keywords:** Accuracy, Shoulder injection, Superomedial approach, Neviaser

## Abstract

*Introduction*: We aimed to compare the accuracy between the standard anterior technique of shoulder injection and the new superomedial technique modified from Neviaser arthroscopic portal placement. Intra-articular placement, especially at the long head of biceps (LHB) tendon, and needle depth were evaluated.

*Methods*: Fifty-eight patients (ages 57 ± 10 years) requiring shoulder arthroscopy in the beach-chair position were recruited. Needle punctures for both techniques were performed by an experienced sports medicine orthopedist. Patients were anesthetized, and the shoulder placed in the neutral position. A single needle was passed through the skin, with only one redirection allowed per trial. The superomedial technique was performed, then the anterior technique. Posterior-portal arthroscopy determined whether needle placement was inside the joint. The percentage of intra-articular needle placements for each technique defined accuracy. When inside the joint, the needle’s precise location was determined and its depth measured. A marginal χ^2^ test compared results between techniques.

*Results*: The superomedial technique was significantly more accurate than the anterior technique (84% vs. 55%, *p* < 0.05). For superomedial versus anterior attempts, the LHB tendon was penetrated in 4% vs. 28% of patients, respectively, and the superior labrum in 35% vs. 0% of patients, respectively; the needle depth was 42 ± 7 vs. 32 ± 7 mm, respectively (all *p* < 0.05).

*Conclusions*: The superomedial technique was more accurate, penetrating the LHB tendon less frequently than the standard anterior technique. A small-diameter needle was needed to minimize superior labral injury. The superomedial technique required a longer needle to access the shoulder joint.

## Introduction

Shoulder aspiration and injection are routine diagnostic and therapeutic procedures for patients with shoulder problems such as stiffness or arthritis. Intra-articular shoulder injection currently employs two standard techniques: anterior (or anterosuperior) and posterior. Previous studies have reported that standard shoulder injection has variable accuracy (based on the percentage of intra-articular needle placements) [1–9] and a high rate of penetration of the LHB tendon [5, 6].

The anterior technique was reported to have an accuracy of 27–65% (in awake subjects) with a single needle pass [1, 7], 95–96% (in cadavers) [4, 5], and 91–100% (in anesthetized subjects) with needle repositioning [6, 9]. This variation was the result of differences in research methodology – e.g., using formalin-fixed or fresh cadavers; awareness of the subjects; number of needle repositionings allowed; and sensation of resistance before successful needle placement. Reportedly, the anterior technique penetrates the LHB tendon in 17–24% of attempts [5, 6]. However, most of these previous studies did not have the comparison groups.

Kim et al. studied in cadavers (supine position) using the superior approach. The entry site was in front of the acromioclavicular (AC) joint. Thus, it could be classified as the anterior approach. The shoulders were in 5°–10° of internal rotation to avoid the LHB tendon. They reported that the LHB tendons were penetrated in 3 of 18 shoulders (16.6%) [5]. There are some doubts about the shoulder position they chose, which was suggested to save the LHB tendon.

Johnson et al. assessed the accuracy of the anterosuperior shoulder injection in 42 anesthetized patients in beach-chair position with the arm in adduction and internal rotation. They reported that the accuracy rate was 91% while the LHB tendons were penetrated in 24%. In their discussion, they recommended positioning the arm in 20°–30° external rotation to move the LHB tendon away from the plane of the needle insertion [6]. Although this recommended arm position has not been confirmed to avoid LHB tendon injury, their approach is commonly used as the anterior approach in the office settings.

In this study, we investigated a new superomedial injection technique. It was modified from the Neviaser portal used for shoulder arthroscopy [10]. However, we applied it first in a cadaveric pilot study to obtain the correct protocol. We did not include the posterior technique in our study based on the finding that the posterior approach was less accurate than the anterior approach. Sethi and El Attrache studied in 20 cadavers (upright position) per approach and reported that the posterior technique was only 50% accurate, whereas the anterior technique was 80% accurate [2].

The primary objective of this study was to compare the accuracy of shoulder injections between the standard anterior and new superomedial injection techniques. The secondary objectives were to determine intra-articular needle placement in order to evaluate the safety of the intra-articular structures, especially the LHB tendon, and to measure the needle depth for each technique. We hypothesized that the new technique would be more accurate and cause less injury to the LHB tendon than the standard anterior technique. However, it might require a longer needle to access the shoulder joint.

## Materials and methods

**Figure 1.**
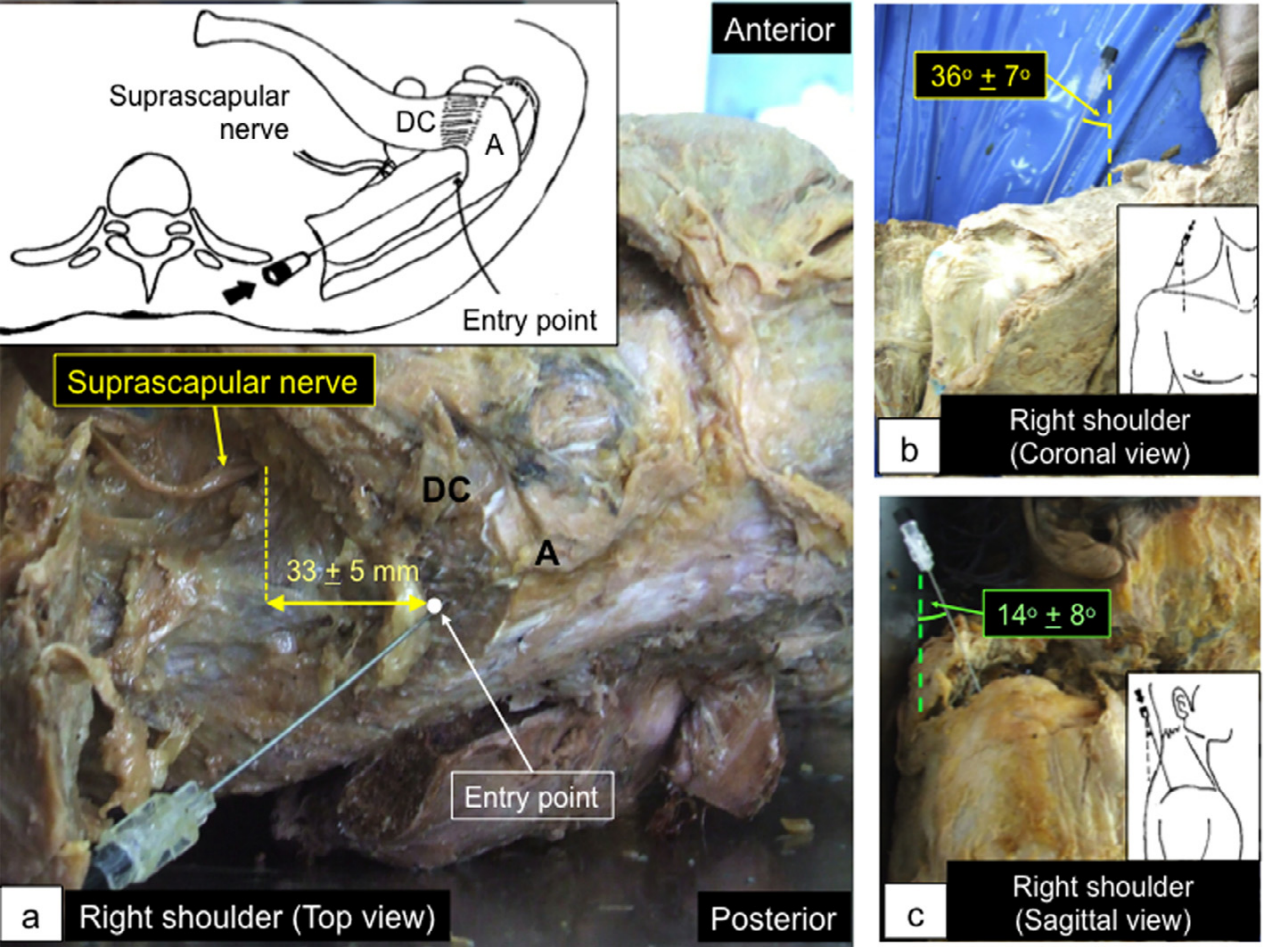
Cadaveric study of the superomedial protocol. (a) The entry point (*white dot*) was about 1 cm posterior to the distal clavicle and just medial to the acromion. It was about 3 cm away from the suprascapular nerve along the mediolateral axis. (b) and (c) The needle angle was 36° ± 7° lateral in the coronal plane (b) and 14° ± 8° anterior in the sagittal plane (c). A: acromion; DC: distal clavicle.

First, we performed the cadaveric pilot study in 16 shoulders of eight cadavers to establish a proper protocol for the superomedial technique. The cadavers were in the supine position. A 22-gauge 90-mm-long (3.5 inches) spinal needle was inserted into the glenohumeral joint using an approach that was modified from the Neviaser arthroscopic portal placement. The needle was repositioned until it entered the joint. The intra-articular needle placement was confirmed by anterior arthrotomy. Results showed that the entry point was about 1 cm posterior to the distal clavicle, just medial to the acromion. It was about 3 cm along the mediolateral axis away from the suprascapular nerve (Figure 1). A goniometer was used to measure the angles of the needle, referenced with the longitudinal axis of the body, in both coronal and sagittal planes. The optimal needle angles for joint access were determined to be 36° ± 7° laterally in the coronal plane and 14° ± 8° anteriorly in the sagittal plane (Figure 1). This protocol would be further used in our participants.

 This study was approved by our Institutional Ethics Committee (Project Number: MTU-EC- OT-0-072/55). From July 2011 to July 2012, patients at Thammasat University Hospital who met the inclusion criteria were enrolled. Inclusion criteria were age > 18 years and planned shoulder arthroscopy in the beach-chair position. Exclusion criteria were a massive rotator cuff tear, shoulder instability (unidirectional or multidirectional), and post-traumatic shoulder injury (previous fracture or dislocation around the shoulder, such as fracture of the distal clavicle, scapula, or proximal humerus, acromioclavicular dislocation). Patients underwent routine, preoperative shoulder radiography depending on their diagnosis. Magnetic resonance imaging (MRI) of the shoulders was performed as needed. Diagnoses included subacromial impingement or bursitis, rotator cuff tear, adhesive capsulitis, and calcific tendinosis. Informed consent was obtained from each participant in the study.

In each case, the patient was brought to the operating room, and general anesthesia was induced. Beach-chair positioning was performed. The operated shoulder was placed in neutral position. Needle punctures were performed by a single skilled orthopedic surgeon with approximately 15 years of experience in orthopedic sports medicine. A single needle pass through the skin, with only one redirection, was allowed for each puncture, according to Johnson et al. [6]. The superomedial puncture was performed first, followed by the anterior puncture.

**Figure 2.**
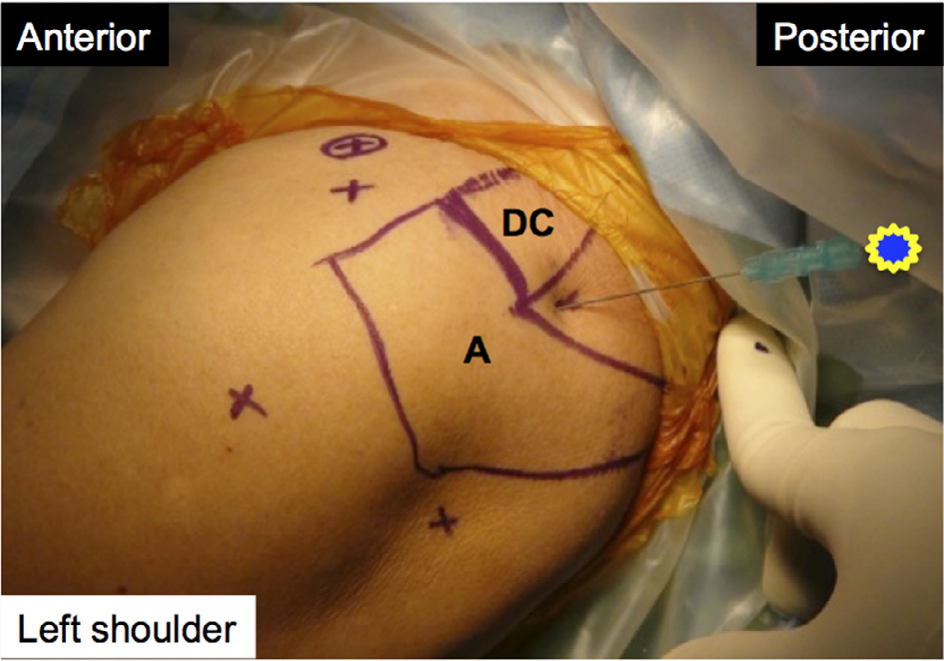
Superomedial technique. A: acromion; DC: distal clavicle.

**Figure 3.**
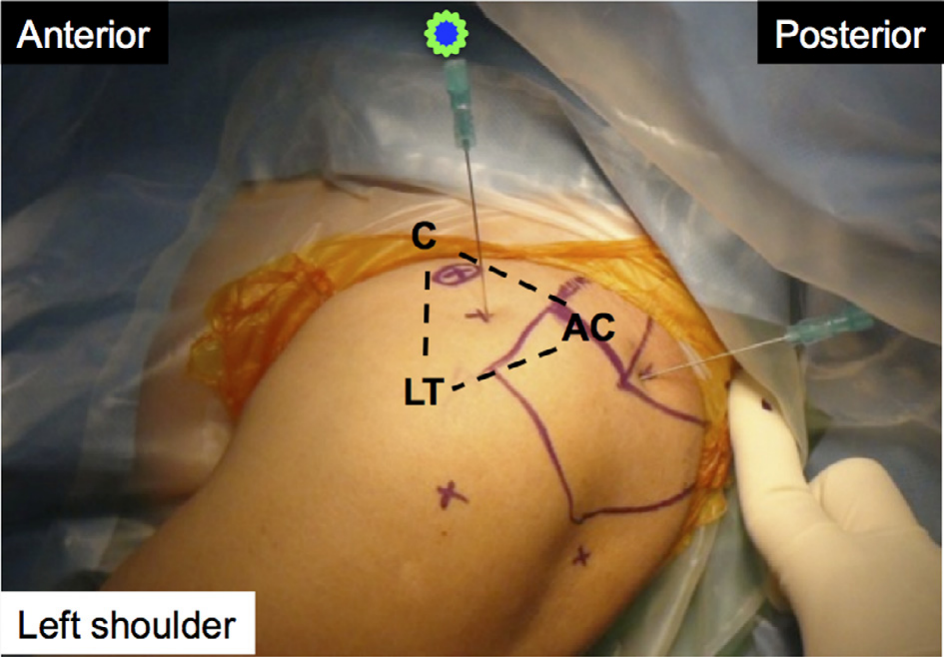
Anterior technique. AC: acromioclavicular joint; C: coracoid; LT: lesser tubercle.

For the superomedial technique, a 23-gauge 90 mm long (3.5 inches) spinal needle was inserted about 1 cm posterior to the distal clavicle and just medial to the acromion. The needle angle was about 30° laterally and 15° anteriorly (Figure 2), according to the results of our cadaveric pilot study. For the anterior technique, we inserted the same-size needle in the same shoulder. The entry point was in the triangular area between the acromioclavicular joint, coracoid process of the scapula, and lesser tubercle of the humerus. The needle was angled about 45° caudally, according to Johnson et al. [6] (Figure 3).

**Figure 4.**
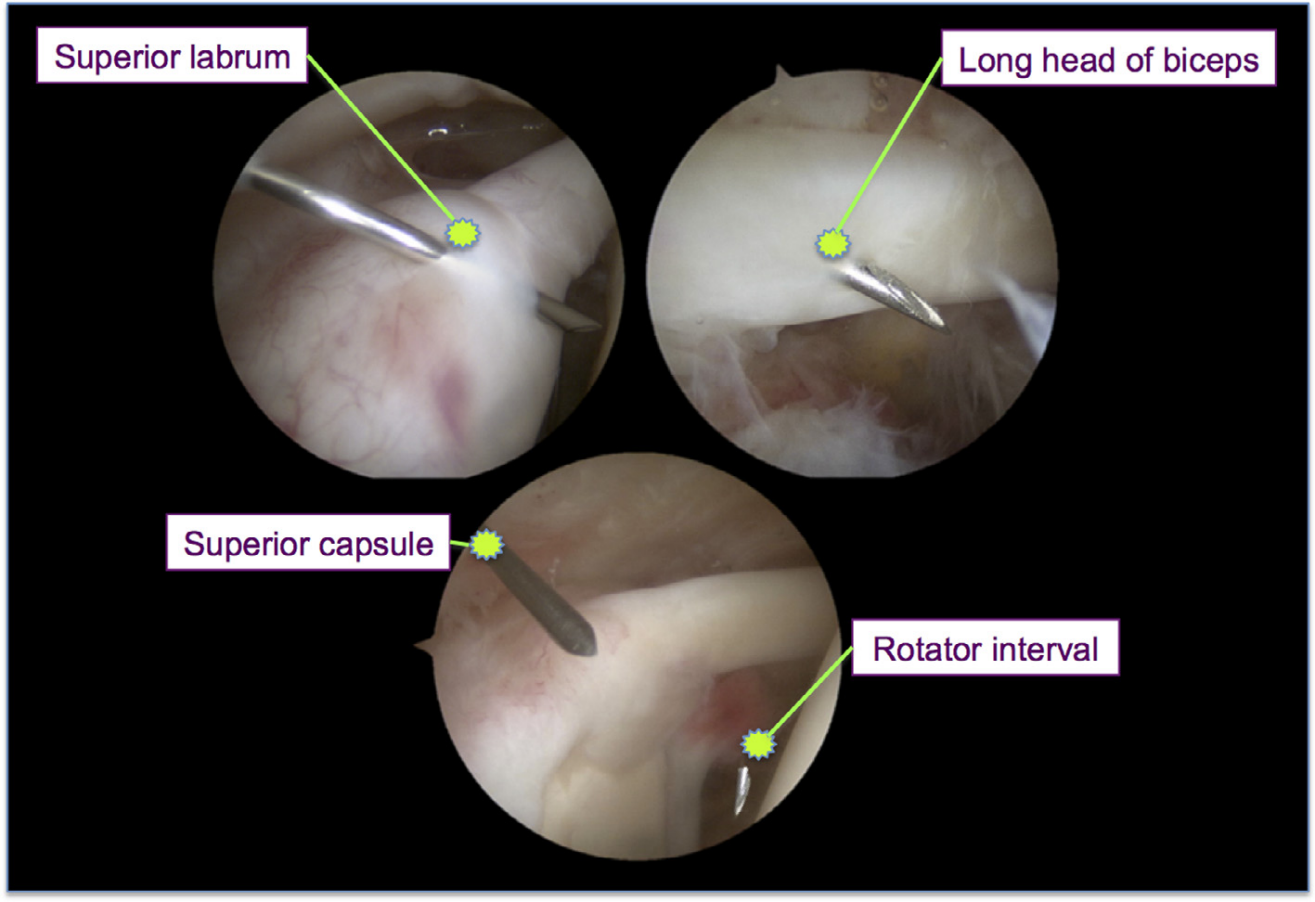
Determination of needle location from arthroscopic findings.

 Standard posterior-portal arthroscopy was performed to assess the needle positions within the shoulder joint. Normal saline was used to fill the joint space for a clear arthroscopic view. Because this step could partially alter the joint capsule and surrounding soft tissue, we inserted all needles before performing single posterior-portal arthroscopy. Under arthroscopy, the needle was first determined to be inside or outside the joint. If inside, it was recorded as intra-articular. The accuracy of the procedure was determined according to the percentage of intra-articular needles after a single needle pass allowing for one redirection. The exact needle location was also determined: in the superior capsule, superior labrum, rotator interval, LHB tendon, or subscapularis muscle (Figure 4). Afterward, the percentages of each joint structure’s penetration were calculated for both techniques. The depth of the needle (i.e., the distance from the skin’s outer surface to the inner surface of the joint capsule) was then measured. After the tip of the needle was moved backward to reach the inner joint capsular surface, an arterial clamp was used to grasp the needle close to the skin outer surface. The needle was then removed from the shoulder, and the distance from the arterial clamp to the tip of the needle was measured.

## Statistical analysis

Sample size calculation for a paired design determined that at least 48 shoulders per technique were required to detect a difference in accuracy of >10% with 80% power. Both techniques were performed in each patient. Descriptive statistics were used for demographic data. Quantitative data were expressed as the means ± *SD* and qualitative data as numbers and percents. The accuracies of the anterior and superomedial techniques were compared using the marginal χ^2^ test. This test was also used to compare the percentages of penetration of each joint structure between the two techniques. The paired Student’s *t*-test was used to compare the depth of needles for the two techniques. A value of *p* < 0.05 was considered to indicate statistical significance. Statistical analysis of the results was conducted using SPSS for Windows, version 10.0 (SPSS, Inc., Chicago, IL, USA).

## Results

In all, 58 patients met the inclusion criteria and were enrolled in the study. The ratio of male (*n* = 23) to female (*n* = 35) patients was about 2:3. The mean ± *SD* age was 57 ± 10 years (range, 34–80 years). The mean ± *SD* body mass index was 24.5 ± 4.0 kg/m^2^ (range, 17.3–36.3 kg/m^2^). The right shoulder was affected in 68% of patients. The most frequent diagnoses were subacromial impingement (91%), rotator cuff tear (66%), and biceps tendinosis (45%), followed by adhesive capsulitis (21%), acromioclavicular arthritis (17%), calcific tendinosis (9%), superior labral tear (2%), and subacromial loose body (2%). Multiple co-morbidities often occurred in the same patient.

Accuracy was significantly greater with the superomedial technique (84%) than with the anterior technique (55%) (*p* < 0.05). Penetration rates of each joint structure were also significantly different between the two techniques, except at the subscapularis muscle (Table 1). The needle tip penetrated the LHB tendon in 28% of attempts with the anterior technique and in only 4% of attempts with the superomedial technique. However, the superomedial technique penetrated the superior labrum in 35% of attempts.

**Table 1.** Summary of accuracy, penetration, and depth of needle.

Outcome	Anterior technique	Superomedial technique
Accuracy^a^	55%	84%*
Penetration sites		
Rotator interval	69%	4%*
Long head of biceps tendon	28%	4%*
Subscapularis	3%	–
Superior capsule	–	57%*
Superior labrum	–	35%*
Depth of needle	32 ± 7 mm	42 ± 7 mm*

a Based on the percentage of intra-articular needle placements.

* *p* < 0.05 (comparison between anterior and superomedial techniques).

Overall needle depth was 42 ± 7 mm for the superomedial technique and 32 ± 7 mm for the anterior technique. This difference was statistically significant (*p* < 0.05). Neither the musculocutaneous nerves nor the suprascapular nerves were injured in this study.

## Discussion

 The accuracy of standard shoulder injections has varied in previous studies because of differences in research methodologies, 27–100% for anterior technique [1–9] and 46–50% for posterior technique [2, 7]. However, most of the previous studies did not have the comparison groups.

Tobola et al. compared the accuracies of anterior, posterior, and supraclavicular (adapted from Neviaser portal placement) techniques in 106 awake patients (33–35 patients per technique). The diagnosis of shoulder was adhesive capsulitis in 54.7% and osteoarthritis in 19.8%. Their results were measured under post-contrast fluoroscopy by one blinded musculoskeletal radiologist. The authors found that the anterior approach (64.7%) tended to be more accurate than the posterior (45.7%) and supraclavicular (45.5%) approaches but that the difference was not statistically significant. However, there were three experienced physicians who chose their preferred approach, and three less-experienced physicians whose approaches were randomized [7]. Thus, there was bias in allocation of approaches. Their sample sizes, which amounted to 13–20 subjects per physician and two physicians per approach, may be too small to enable an adequate comparison.

Our results demonstrated that the superomedial technique was significantly more accurate than the anterior technique (84% vs. 55%). This could be used as an alternative approach for shoulder aspiration or injection in the clinical setting. It also had a significantly lower rate of LHB tendon penetration than the anterior technique (4% vs. 28%). This could be the result of differing entry points (i.e., posterior to the AC joint with the superomedial technique but anterior to it with the anterior technique). The rate of penetration for the anterior technique was similar to the results of previous studies (17% [5] and 24% [6]).

Tendinopathy, dislocation, and partial or complete tears can be found in the LHB tendon, particularly in patients with a rotator cuff tear (up to 90%) or glenohumeral arthritis [11]. Most patients with a pathologic LHB tendon, except with the complete-tear condition, have already had anterior shoulder pain [12]. Therefore, if this pathologic tendon is penetrated, it tends to become worse because of enlarging the tear and the patients tend to experience more pain during the injection. For these reasons, it is important to use the injection technique that is least likely to injure this tendon.

 In this study, small-diameter (23-gauge) needles were used to decrease the severity of any injuries. The superior labrum was penetrated significantly more often with the superomedial technique than with the anterior technique (35% vs. 0%). However, this factor may not have great clinical significance because the glenoid labrum is composed of fibrocartilage, which mainly helps stabilize the shoulder joint. It is not hyaline or articular cartilage, which smooths the articulation and provides a low-friction gliding joint surface. Additionally, the area penetrated by the needle was small compared with that of the labrum.

This study had some limitations. First, all patients who were to undergo shoulder arthroscopy in the beach-chair position and met the inclusion criteria were enrolled. Therefore, we included patients other than those who required shoulder injection or aspiration as outpatients for conditions such as osteoarthritis, rheumatoid arthritis, or adhesive capsulitis of the shoulders [13]. In this study, 21% of the patients had adhesive capsulitis. There were no cases of osteoarthritis or rheumatoid arthritis of the shoulders. Second, no blinding was performed in regard to needle placement and evaluation, which were performed by the same person. Finally, because patients were anesthetized, muscular and emotional responses to the puncture may have differed from those in awake patients.

## Conclusions

This study showed that, for shoulder injection, the superomedial technique was more accurate and had a lower rate of penetration of the LHB tendon than the anterior technique. However, a small-diameter needle was needed to minimize injury to the superior labrum. Additionally, the new technique requires a longer needle, including spinal needle, to access the shoulder joint. Future studies specific to patients with shoulder osteoarthritis, rheumatoid arthritis, or adhesive capsulitis, and those who require shoulder injection or aspiration at orthopedic outpatient departments, are needed.

## Conflict of interest

BC and NZ certify that they have no conflict of interest.
